# Plasma and urinary concentrations of arachidonic acid-derived eicosanoids are associated with diabetic kidney disease

**DOI:** 10.17179/excli2021-3408

**Published:** 2021-03-18

**Authors:** Sonia Mota-Zamorano, Nicolás R. Robles, Juan Lopez-Gomez, Bárbara Cancho, Luz M. González, Guadalupe Garcia-Pino, María Luisa Navarro-Pérez, Guillermo Gervasini

**Affiliations:** 1 Department of Medical and Surgical Therapeutics, Division of Pharmacology, Medical School, University of Extremadura, Badajoz, Spain; 2 Service of Nephrology, Badajoz University Hospital, Badajoz, Spain; 3 Service of Clinical Analyses, Badajoz University Hospital, Badajoz, Spain; 4 Service of Nephrology, Zafra Hospital, Badajoz, Spain; 5 Department of Biomedical Sciences, University of Extremadura, Badajoz, Spain

**Keywords:** 20-HETE, DHETs, Diabetic Kidney Disease, EETs, eicosanoids

## Abstract

Preclinical studies indicate that arachidonic acid (AA)-derived eicosanoids contribute to hyperglycemia-induced kidney injury. We aimed to determine whether plasma and/or urinary levels of dihydroxyeicosatrienoic (DHETs) and 20-hydroxyeicosatetraenoic (20-HETE) acids are associated with diabetic kidney disease (DKD). A total of 334 subjects (132 DKD patients and 202 non-diabetic individuals) were studied. Plasma levels of 11,12-DHET, 14,15-DHET and 20-HETE were measured by LC/MS/MS. Urinary 20-HETE concentrations were determined by immunoenzymatic assay. Subjects with normoalbuminuria had larger 20-HETE-to-creatinine urinary ratios (20-HETE/Cr) than those with micro and macroalbuminuria (p=0.012). Likewise, participants with eGFR>60 ml/min/1.73 m^2^ had higher plasma levels of 14,15-DHET (p=0.039) and 20-HETE/Cr ratios (p=0.007). Concentrations of 14,15-DHET, 11,12-DHET and 20-HETE/Cr were significantly lower in DKD patients. Median values for non-diabetic vs. DKD were, respectively, 493 (351.0-691.5) vs. 358 (260.5-522) ng/L, p=3e-5; 262 (183.5-356.0) vs. 202 (141.5-278.0) ng/L, p=1e-4 and 5.26 (1.68-11.65) vs. 2.53 (1.01-6.28) ng/mgCr, p=0.010. In addition, 20-HETE/Cr ratios were higher in patients with non-proteinuric DKD than in those with typical DKD (p=0.020). When only individuals with impaired filtration were considered, 14,15-DHET and 11,12-DHET levels were still higher in non-diabetic subjects (p=0.002 and p=0.006, respectively). Our results indicate that AA-derived eicosanoids may play a relevant role in DKD.

## Introduction

Diabetic kidney disease (DKD) is one of the main complications of diabetes mellitus and the most common cause of end-stage renal disease (Ahmad, 2015[1]). It affects roughly 40 % of patients diagnosed with diabetes (Gnudi et al., 2016[16]), contributing significantly to their morbidity and mortality. Consequences derived from DKD include decreased glomerular filtration rate, increased albumin levels, proteinuria, fluid retention, elevated arterial blood pressure and renal failure (Ahmad, 2015[1]). Proteinuria has traditionally been considered the most important biomarker of the disease; however, it has been recently pointed out that a significant percentage of patients with diabetes present with a decreased renal function without proteinuria (nonproteinuric diabetic kidney disease). There is therefore an increasing need of finding new effective and representative markers of DKD (Robles et al., 2015[31]; Yamanouchi et al., 2020[38]).

In the last years, some studies have pointed to the epoxygenase pathway of arachidonic acid (AA) metabolism as a route that might be deeply involved in the mechanisms underlying DKD. AA is biotransformed by cytochrome P (CYP) 450 enzymes into different vasoactive eicosanoids. Epoxieicosatrienoic acids (EETs) are one of these mediators, which have vasodilator, antinflammatory and, in general, renoprotective properties (Yang et al., 2015[39]; He et al., 2017[17]; Huang et al., 2018[20]). These are however highly unstable compounds that are rapidly biotransformed by soluble epoxyhydrolase (sEH) into more stable, but significantly less active, dihydroxy-eicosatrienoic acids (DHETs) (Deng et al., 2010[4]). Another important AA-derived eicosanoid is 20-hydroxyeicosatetraenoic acid (20-HETE), which is excreted in urine as a glucuronide conjugate (Prakash et al., 1992[30]), and that is an important regulator of vascular and renal functions (Hoopes et al., 2015[19]; Wang et al., 2019[36]). 20-HETE actions are quite complex; for instance, this mediator can exert both prohypertensive and antihypertensive actions (Gangadhariah et al., 2015[12]).

Previous reports have shown that, while EETs may prevent hyperglycemia-induced damage in the kidney (Hoff et al., 2019[18]; Jiang et al., 2020[23]), 20-HETE seems to have detrimental effects (Eid et al., 2009[7], 2013[8][9]; Luo et al., 2009[25]; Ding et al., 2019[5]). However, to date, these studies have all been carried out *in vitro* or in animal models and there are no clinical studies that can help understand the real involvement of these AA metabolites in DKD. In the present work, we aim to determine whether the plasma and urinary levels of these vasoactive eicosanoids may be indicative of the presence of the disease and/or correlate with relevant parameters of renal function.

## Patients and Methods

### Study subjects

The study included 334 Caucasian patients over 18 years of age treated at the Services of Nephrology and the Hypertension Units of three different hospitals in the province of Badajoz (Spain) between June 2017 and December 2019. After the participants gave written informed consent for their enrolment, blood (10 ml) and urine (18 ml) samples were collected. Blood samples were then centrifuged to obtain plasma, which was stored at -80 ºC together with the urine until analysis.

**Figure 1:**
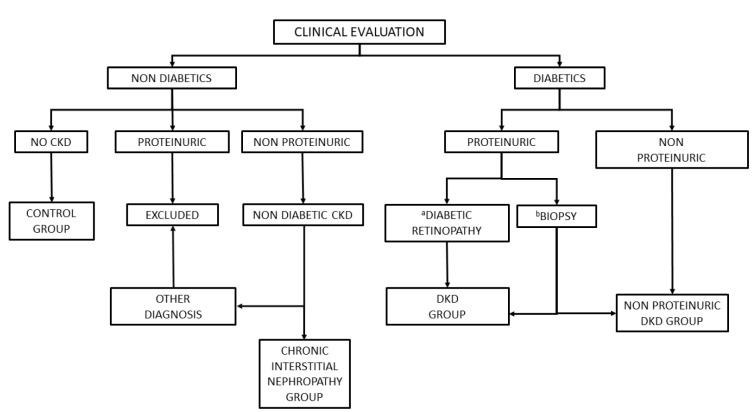
Diagnosis algorithm. CKD, chronic kidney disease; DKD, diabetic kidney disease ^a^With negative immunological markers, ^b^No diabetic retinopathy or positive immunologic markers

DKD diagnosis was carried out according to the algorithm shown in Figure 1(Fig. 1), which further stratified patients into those with proteinuric (typical) and non proteinuric DKD. Non-diabetic individuals were also stratified into those with and without chronic kidney disease (CKD). Patients in the DKD group had to have Type 2 diabetes (fasting glucose > 126 mg/dL or non-fasting glucose > 200 mg/dL) before the presence of kidney disease, defined as albuminuria or eGFR < 60 mL/min. Overt albuminuria was defined as having > 300 mg albumin excreted in urine over 24 hours, whilst values from 30 to 300 mg were considered as microalbuminuria. Typical DKD was diagnosed by biopsy or by clinical criteria, namely the presence of both retinopathy and albuminuria after exclusion of other possible causes. Other possible renal diseases were excluded using standard diagnostic protocols. In cases in which proteinuria was higher than 1 g/day, a renal biopsy was performed to confirm the diagnosis, provided the patient had given his/her consent. Non proteinuric DKD was diagnosed in the presence of reduced glomerular filtration without proteinuria in diabetic patients after excluding other renal diseases.

The study protocol was approved by the Ethics Committee of the University Hospital of Badajoz and it was carried out in accordance with the Declaration of Helsinki and its subsequent revisions.

### Analysis of plasma eicosanoid levels

One 0.5-ml plasma aliquot was used for the quantitative determination of the different eicosanoids, namely 20-HETE, 14,15-DHET and 11,12-DHET. These DHETs are the product of a very rapid metabolic transformation of vasoactive EETs by the soluble epoxy hydrolase and their quantification was used as a surrogate of the corresponding EETs levels as previously described (Spiecker et al., 2004[33]; Yang et al., 2013[40]). Prior to quantification, plasma samples were processed by solid phase extraction using Hypersep Retain Pep 60 mg 3 ml S columns (Thermo Fisher Scientific, Waltham, MA, USA). Separation and measurement of the concentration of the AA metabolites in plasma was performed by mass spectrometry coupled to liquid chromatography (LC/MS/MS) using a UHPLC 1290 system with a 6460 Jet Stream triple quadrupole mass detector (Agilent Technologies, Santa Clara, CA, USA). Details of the chromatographic technique can be found elsewhere (Orozco et al., 2013[29]). A typical chromatogram showing retention times for all the analytes in plasma is shown in Supplementary Figure 1.

### Determination of urinary concentrations of 20-HETE

Free and glucuronidated 20-HETE concentrations in urine were determined by a 20-HETE/beta-glucuronidase competitive immunoenzymatic assay kit according to the manufacturer's instructions (Abcam, Cambridge, UK). Briefly, samples were first digested with beta-glucuronidase for 3 hours in order to allow the determination of conjugated 20-HETE. Next, all samples and standards were diluted as recommended and 1x-HRP conjugate was added to all wells except the blank control wells. After a 2-hour incubation and several washing steps, TMB substrate was added and the plate was once more incubated at room temperature for 30 minutes. The reaction was stopped by the addition of 2N sulphuric acid and the plate was read at 450 nm in a Biotek ELx808 plate reader (Biotek Instruments Inc., Winooski, VT, USA). The intra- and inter-assay variation was tested and found to be less than 10 % and 15 %, respectively. Urine was not available for those DKD patients who were on dialysis at the time of the study and, in consequence, the urinary concentrations of 20-HETE could not be determined in these individuals.

### Statistical analysis

**Figure 2:**
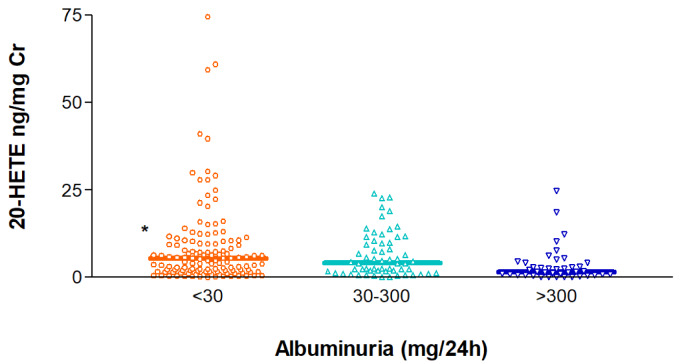
Distribution of the urinary excretion of 20-HETE corrected for creatinine in individuals with normoalbuminuria (< 30 mg/24 h), microalbuminura (30-300 mg/24 h) and macroalbuminuria (> 300 mg/ 24 h). ^*^p=0.001

Data from continuous variables are presented as mean value ± standard deviation or median (interquartile range, IQR) in the case of nonparametric distribution. Categorical variables are presented as count with percentage in parentheses. Associations between categorical variables were analyzed using Fisher's exact test. Comparisons of quantitative data across groups were performed with Mann-Whitney/T-test or Kruskal-Wallis/ ANOVA tests depending on the number of groups and the normality of the data. Differences between study groups, e.g., diabetic vs. non-diabetic subjects, regarding the levels of eicosanoids were evaluated by logistic regression, including in the models relevant covariates, namely age, sex, weight, hypertension, hyperlipidemia and diabetes (the latter only in the analysis of associations with eGFR and proteinuria in the entire cohort). One outlier in Figure 2 and two outliers in Figures 4 and 5 were excluded from the figures in order to improve the visibility of the charts. The threshold for outliers was determined by multiplying the IQR by 1.5 and adding the result to the third quartile. Statistical analyses were carried out using IBM SPSS Statistics package v.22 (IBM Corporation, Armonk, NY, USA). A p-value <0.05 was considered statistically significant.

## Results

Characteristics of the study population are summarized in Table 1(Tab. 1). The study included 132 patients (95 males) with DKD and 202 non-diabetic subjects (117 males). DKD patients were an average of 10 years older than non-diabetic individuals and had a higher incidence of hypertension and hyperlipidemia (Table 1(Tab. 1)).

### Association of eicosanoids levels and parameters of renal function in the whole study sample

20-HETE excretion in urine has been pointed out as a potential indicator of human diseases (Nithipatikom et al., 2006[28]; Minuz et al., 2008[27]), which prompted us to also quantify this AA metabolite in the urine of all the study participants. The urinary 20-HETE-to-creatinine ratio (20-HETE ng/mg Cr) was measured in subjects with normoalbuminuria (< 30 mg/ 24 h), microalbuminuria (30-300 mg/24 h) and macroalbuminuria (> 300 mg/24 h), which revealed statistically significant differences in the observed ratios across the three groups (p=0.012), with higher values in individuals without proteinuria and lower ratios in those with macroalbuminuria. Median (IQR) values were 5.50 (1.90-10.50), 4.16 (1.74-10.15) and 1.54 (0.62-4.16) ng/mg Cr for the three groups (Figure 2(Fig. 2)). Plasma concentrations of 14,15-DHET or 11,12-DHET did not show a significant association with albuminuria (Supplementary Figure S2).

**Figure 3:**
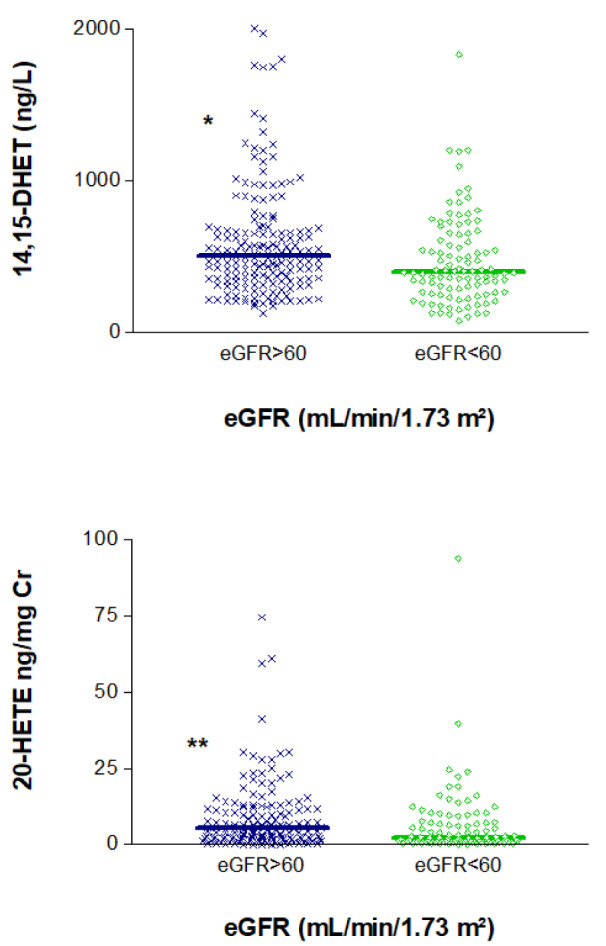
Association of plasma levels of 14,15 DHET and 20-HETE/creatinine ratios in urine with estimated glomerular filtration rate (eGFR). *p<0.05, **p<0.01

We also examined the entire study sample to determine whether the eicosanoids of interest were associated with eGFR. Indeed, we observed significant differences in the levels of these mediators between individuals with eGFR < 60 mL/min/1.73 m² and those with higher values. Median (IQR) values of 14,15-DHET and 20-HETE/Cr in patients with high vs. low eGFR were, respectively, 504 (351-672) vs. 398 (267-627) ng/L, p=0.039 and 5.34 (2.14-11.65) vs. 2.25 (0.99-7.51) ng/mg Cr; p=0.007 (Figure 3(Fig. 3)).

The concentrations of 20-HETE in plasma did not show any associations with parameters of renal function (Supplementary Figure 3).

### Association of eicosanoids levels with diabetic nephropathy

**Figure 4:**
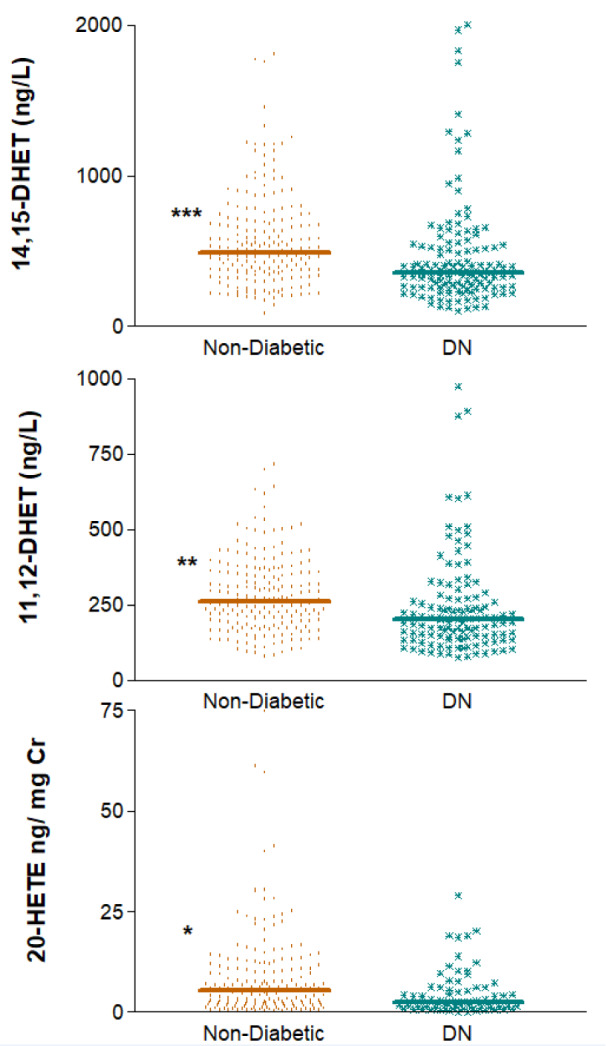
Differences between patients with diabetic nephropathy (DN) and non-diabetic subjects regarding plasma levels of 14,15- and 11,12-DHET and the urinary excretion of 20-HETE corrected for creatinine. DN, diabetic nephropathy ^*^p=0.01, ^**^p<0.001, ^***^p<0.0001

Next, we analyzed whether any of the AA-derived metabolites were indicative of the presence of DKD. Indeed, the results depicted in Figure 4(Fig. 4) show that, after adjusting for relevant covariates, subjects without diabetes had significantly higher levels of 14,15-DHET, 11,12-DHET and 20-HETE/Cr than patients with DKD did. Median values (IQR) displayed for non-diabetic vs. diabetic subjects for the three eicosanoids were, respectively, 493 (351-691.5) vs. 358 (260.5-522) ng/L, p=3 e-5; 262 (183.5-356) vs. 202 (141.5-278) ng/L, p=1 e-4 and 5.26 (1.68-11.65) vs. 2.53 (1.01-6.28) ng/mg Cr, p=0.010 (Figure 4(Fig. 4)).

**Figure 5:**
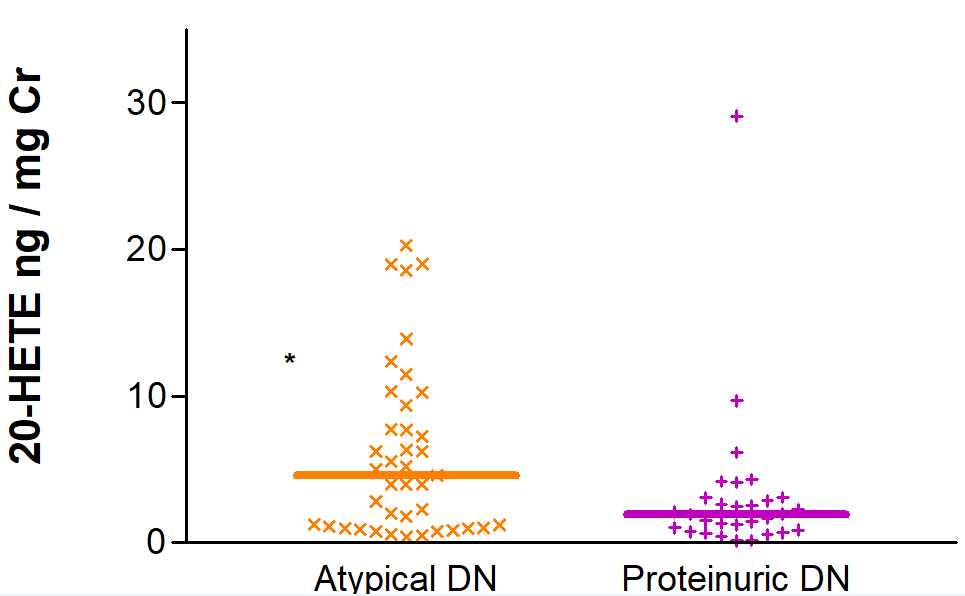
Urinary excretion of 20-HETE corrected for creatinine in patients with overt and atypical diabetic nephropathy (DN). ^*^p<0.05

Interestingly, when the DKD group was studied in isolation, 20-HETE/Cr ratios were found to be significantly elevated in patients with atypical DKD in comparison with diabetic patients presenting with overt proteinuria. Median (IQR) values for the two groups were 4.55 (1.06-9.81) vs. 1.95 (0.80-3.09) ng/mg Cr, p=0.020 (Figure 5(Fig. 5)).

**Figure 6:**
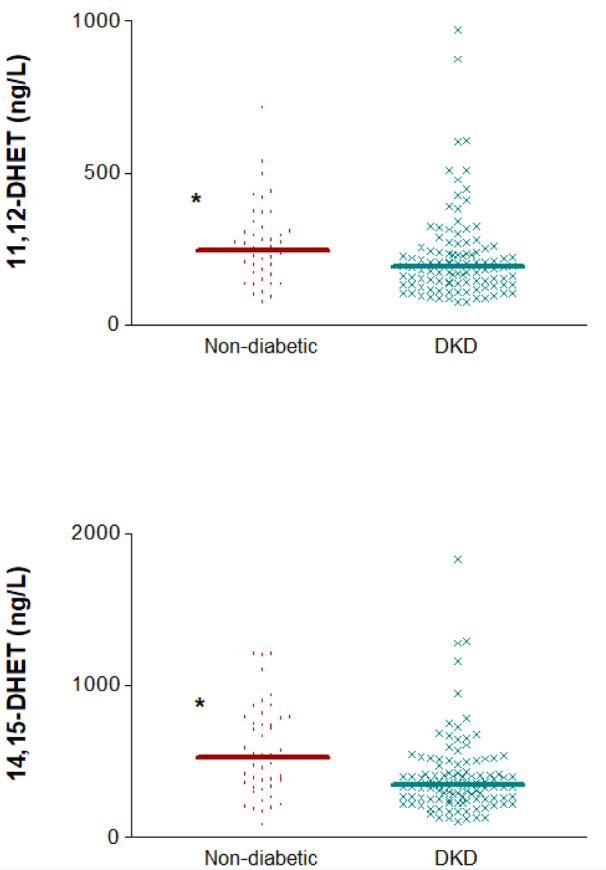
Distribution of 14,15- and 11,12-DHET plasma concentrations in diabetic (DKD) and non-diabetic subjects with impaired glomerular filtration. ^*^p<0.01

Finally, the concentrations of these AA-derived eicosanoids were compared between DKD patients and a subset of non-diabetic subjects who all had low eGFR (below 60 mL/min/1.73 m²). There were no significant differences regarding eGFR between both groups (p=0.162), as median (IQR) values were very similar: 37.35 (28.95-47.00) for DKD and 39.09 (32.48-51.50) ml/min/1.73 m^2^ for individuals without diabetes. The levels of 14,15-DHET and 11,12-DHET and in plasma were significantly higher in the non-diabetic subjects [529 (321.5-780.0) vs. 346.5 (254.5-453.0) ng/L, p=0.002, for 14,15-DHET and 246 (173.5-311.0) vs. 191.5 (135.0-259.0) ng/L, p=0.006, for 11,12-DHET] (Figure 6(Fig. 6)). The raw data used to generate Figures 2 to 6(Fig. 2)(Fig. 3)(Fig. 4)(Fig. 5)(Fig. 6) are presented in Supplementary Table S1.

## Discussion

There is a growing body of evidence pointing to AA-derived vasoactive eicosanoids as important players in the cardiorenal function (Gervasini et al., 2015[13][14], 2018[15]; Fang et al., 2018[10]; Imig, 2019[22]). These findings, together with the existence of preclinical data linking these AA metabolites to the renal damage induced by hyperglycemia (Luo et al., 2009[25]; Eid et al., 2013[9]), suggest that the levels of these eicosanoids could be useful indicators of the presence of DKD in renal patients, a hypothesis we test in the present work.

With regard to the analysis of parameters of renal function, our results show that the urinary excretion of 20-HETE corrected by creatinine, was far lower in individuals with albuminuria. Consistent with our findings, Satarug et al. showed that albuminuria was associated with decreased urinary 20-HETE concentrations in men chronically exposed to cadmium, a risk factor for CKD (Satarug et al., 2019[32]). If we assume that a lower urinary excretion implies higher endogenous levels of 20-HETE, these findings would point to a damaging role of 20-HETE in kidney disease. To our knowledge, there are no more clinical studies examining the association of 20-HETE with proteinuria; however, and in contrast to our results, some animal models have reported that 20-HETE in the glomerulus helps maintain glomerular filtration barrier to albumin (McCarthy et al., 2005[26], Williams et al., 2007[37]). It should be noted though, that these were not diabetic models. The intricate functions of 20-HETE in the renal tubules and vasculature may be behind these conflicting results. In fact, it has been suggested that 20-HETE can play opposite roles on kidney homeostasis depending on the cell type that produces and/or targets this AA-derived lipid (Gangadhariah et al., 2015[12]).

The other index of renal function, eGFR, also showed a significant association with 20-HETE/Cr ratios in urine, as the excretion of this eicosanoid in our study sample was significantly higher in individuals with eGFR > 60 mL/min/1.73 m². Albeit this is the first DKD study measuring levels of vasoactive eicosanoids, Dreisbach et al. also showed that 20-HETE/Cr levels in urine positively correlated with eGFR in CKD African-American patients with varying etiologies (Dreisbach et al., 2014[6]). Therefore, the analysis of both proteinuria and eGFR in our study suggest that a lower excretion of 20-HETE is associated with poorer renal function. Dreisbach et al. argued, and we agree, that this observation might obey to a decrease in the filtration of this mediator but also to the fact that 20-HETE may play a prominent role in the pathophysiology of renal damage induced by hyperglycemia (Dreisbach et al., 2014[6]).

The analysis of the differences between DKD patients and non-diabetic individuals regarding the 20-HETE/Cr ratio revealed a significantly lower excretion in patients with DKD compared with non-diabetic individuals. This, and the aforementioned associations with eGFR and proteinuria, all indicate that an accumulation of this eicosanoid (suggested by the observed lower excretion) in tissues where it is highly expressed such as the kidney (Lasker et al., 2000[24]), could constitute a significant contribution to renal injury in diabetic nephropathy, as various *in vitro* studies and animal models have previously proposed (Eid et al., 2009[7], 2013[8];[9] Ding et al., 2019[5]). Indeed, it has been reported that a reduction in renal 20-HETE biosynthesis or the administration of 20-HETE antagonists both protect mice from diabetic-mediated renal injury (Gangadhariah et al., 2015[12]). According to preclinical reports, the mechanism behind the 20-HETE-induced damage in renal tissue would most likely involve promoting hypertension, high glucose-mediated podocyte apoptosis or tubular hypertrophy (Eid et al., 2009[7]; Gangadhariah et al., 2015[12]). We also observed that 20-HETE/Cr urinary levels were also different between DKD patients with overt proteinuria and those with the so-called atypical DKD, whose ratios were 2.5-fold higher. The explanation for this finding most likely lies in the association between 20-HETE and albuminuria that we found for the entire study sample, as patients with atypical DKD usually present with a nonproteinuric phenotype. This observation is interesting because the quantification of 20-HETE excretion might therefore be useful to discriminate patients with proteinuria due to glomerular inflammatory diseases, thus reducing the need for performing biopsies.

EETs synthesized by the cytochrome P-450, particularly 14,15-EET and, to a lesser extent, 11,12-EET, possess antihypertensive properties and have been shown to be endothelium-derived hyperpolarizing factors in the kidney, as well as being anti-inflammatory mediators that protect kidney vasculature in cardiorenal diseases (Imig, 2005[21]). We observed that plasma concentrations of DHETs, EETs direct metabolites via sEH-mediated degradation, were significantly lower in subjects with impaired glomerular filtration rate. To date, the focus of most clinical studies evaluating the putative role of EETs has been put on the cardiovascular setting (Theken et al., 2012[34]; Fava and Bonafini, 2018[11]; Imig, 2019[22]) and, to our knowledge, there are no reports assessing their correlation with eGFR in renal patients. Interestingly enough, however, our group has previously reported an indirect observation in the same line as the results presented herein. We showed how renal transplant recipients carrying a genetic polymorphism that leads to an increased expression of sEH, and hence to a faster degradation of EETs, had lower eGFR values compared to those found in patients with normal sEH expression (Gervasini et al., 2015[13]).

Mirroring the results obtained for 20-HETE, 14,15- and 11,12-DHET plasma levels were also significantly lower in the DKD patients compared with those found in non-diabetic individuals. In the absence of previous clinical studies to which compare our results, several groups have reported similar findings in other settings. For instance, Luo et al. demonstrated in rat models of DKD that hyperglycemia decreases EETs production in the glomeruli, changes that may be important in causing glomerular damage in the early stage of DKD (Luo et al., 2009[25]). Moreover, an enhanced sEH expression (resulting in low levels of EETs) in murine kidneys under streptozotocin-induced diabetes (Chen et al., 2012[3]; Bettaieb et al., 2017[2]; Jiang et al., 2020[23]) and in cells exposed to hyperglycemia (Jiang et al., 2020[23]) has been repeatedly observed. Indeed, an elevation of EETs levels by inhibition of sEH have been suggested as a potential therapeutic strategy for the amelioration of DKD (Chen et al., 2012[3], Jiang et al., 2020[23]). According to these preclinical data, an sEH inhibition leads to higher concentrations of EETs which, in turn, attenuate renal tubular mitochondrial dysfunction and endoplasmic reticulum stress by restoring autophagic flux, as well as decreasing renal tubular apoptosis (Chen et al., 2012[3]; Jiang et al., 2020[23]). Our findings confirm, for the first time in humans, the importance of these eicosanoids in DKD.

An additional remarkable observation was that when only subjects with impaired glomerular filtration were included in the analysis, 14,15-DHET plasma levels were still significantly different between DKD patients and non-diabetic individuals, with the latter showing higher concentrations. This is interesting because the observed difference in the metabolite levels would not rely on the decreased renal function (similar in both groups), but on the presence of diabetes-induced renal damage, which adds to the aforementioned evidence that these eicosanoids must play a central role in hyperglycemia-induced renal damage. As we mentioned in the case of the urinary excretion of 20-HETE, this observation could be helpful for establishing diagnosis in non-proteinuric diabetic patients.

This study has some limitations, namely its cross-sectional design, which resulted in the enrolment of DKD patients with different progression of the disease; a limited sample size, which somewhat hampers the generalization of the results obtained from the analysis of subsets, i.e. non-diabetic patients with decreased renal function; or the use of surrogates (DHETs) to estimate the levels of the rapidly biotransformed EETs, an approach that we and others (Spiecker et al., 2004[33]; Yang et al., 2013[40]) adopted since the EET peaks detected in plasma were often below the level of quantification of the chromatographic technique.

In summary, our results show, for the first time to our knowledge, that levels of vasoactive eicosanoids in plasma and urine correlate with renal function, as indicated by proteinuria and eGFR. More importantly, there are significant differences regarding these levels between patients with DKD and non-diabetic individuals. Interestingly, these differences were still evident for DHETs when filtration impairment was taken out of the equation and only the diabetic disease was considered. These findings taken together suggest AA-derived metabolites in plasma and/or urine might be useful in DKD diagnosis, a pathology still needed of reliable biomarkers (Thi et al., 2020[35]). Notwithstanding, the analysis of larger cohorts of DKD patients is warranted in order to confirm the results presented herein.

## Supplementary material

Supplementary Figures 1 to 3, and Supplementary Table S1 can be found at 10.6084/m9.figshare.13325705 and 10.6084/m9.figshare.14135081, respectively.

## Acknowledgements

We would like to acknowledge the technical and human support provided by the Service of Elemental and Molecular Analysis at SAIUEx. We also thank the patients who participated in this study. This work has been supported in part by grants PI15/00804 and PI18/00745 from Instituto de Salud Carlos III, Madrid (Spain) and grants GR18007 and IB16014 from Junta de Extremadura, Consejería de Economía e Infraestructura, Mérida (Spain) and Fondo Europeo de Desarrollo Regional ”Una manera de hacer Europa”.

## Disclosure statement

The authors declare that they have no conflict of interest.

## Data availability statement

The datasets generated and/or analyzed during the current study are available in the Figshare repository with the following DOI identifier: 10.6084/m9.figshare.14135081.

## Supplementary Material

Supplementary material

Supplementary data

## Figures and Tables

**Table 1:**
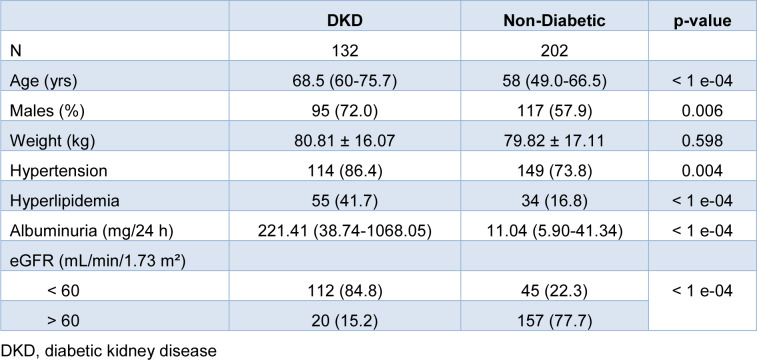
Demographic and clinical characteristics of the study participants. Categorical variables are presented as count (and percentage). Quantitative data are shown as mean ± standard deviation or median (interquartile range), depending on the normality of their distribution.
