# Supplementary material for: Plasma and urinary concentrations of arachidonic acid-derived eicosanoids are associated with diabetic kidney disease
Source: EXCLI J. 2021 Mar 18;20:698–708. doi: 10.17179/excli2021-3408 (PMC8144539; doi:10.17179/excli2021-3408)
Supplement: Supplementary material [file EXCLI-20-698-s-001.pdf]

EXCLI Journal 2021;20:698-708 – ISSN 1611-2156
Received: January 18, 2021, accepted: March 11, 2021, published: March 18, 2021

Supplementary material to:

Original article:

PLASMA AND URINARY CONCENTRATIONS OF ARACHIDONIC ACID-DERIVED EICOSANOIDS ARE ASSOCIATED WITH DIABETIC KIDNEY DISEASE

Sonia Mota-Zamorano[1], Nicolás R. Robles[2], Juan Lopez-Gomez[3], Bárbara Cancho[2], Luz M. González[1], Guadalupe Garcia-Pino[4], María Luisa Navarro-Pérez[5], Guillermo Gervasini[1]*

[1] Department of Medical and Surgical Therapeutics, Division of Pharmacology, Medical School, University of Extremadura, Badajoz, Spain
[2] Service of Nephrology, Badajoz University Hospital, Badajoz, Spain
[3] Service of Clinical Analyses, Badajoz University Hospital, Badajoz, Spain
[4] Service of Nephrology, Zafra Hospital, Badajoz, Spain
[5] Department of Biomedical Sciences, University of Extremadura, Badajoz, Spain

* **Corresponding author:** Guillermo Gervasini, PhD, Department of Medical and Surgical Therapeutics, Division of Pharmacology, Medical School, University of Extremadura, Av. Elvas s/n 06071, Badajoz, Spain. Phone: +34 924 289 824, E-mail: ggervasi@unex.es

http://dx.doi.org/10.17179/excli2021-3408

This is an Open Access article distributed under the terms of the Creative Commons Attribution License (http://creativecommons.org/licenses/by/4.0/).

EXCLI Journal 2021;20:698-708 – ISSN 1611-2156
Received: January 18, 2021, accepted: March 11, 2021, published: March 18, 2021

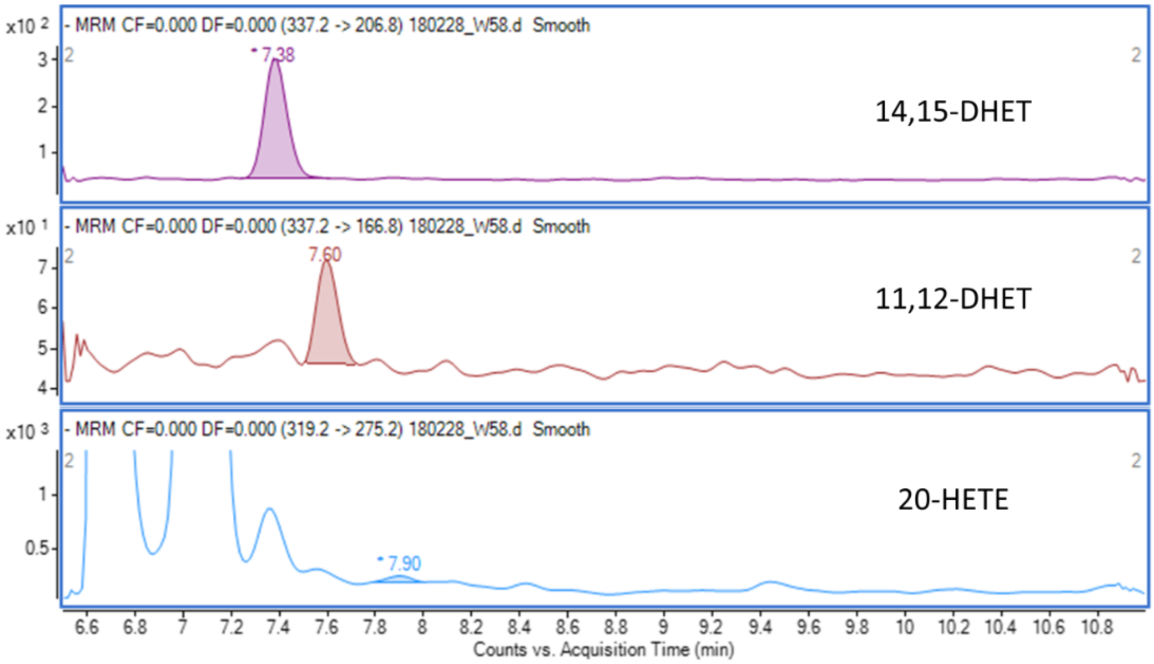

Supplementary Figure 1: Chromatogram showing retention times for the araquidonic-acid metabolites in plasma

EXCLI Journal 2021;20:698-708 – ISSN 1611-2156
Received: January 18, 2021, accepted: March 11, 2021, published: March 18, 2021

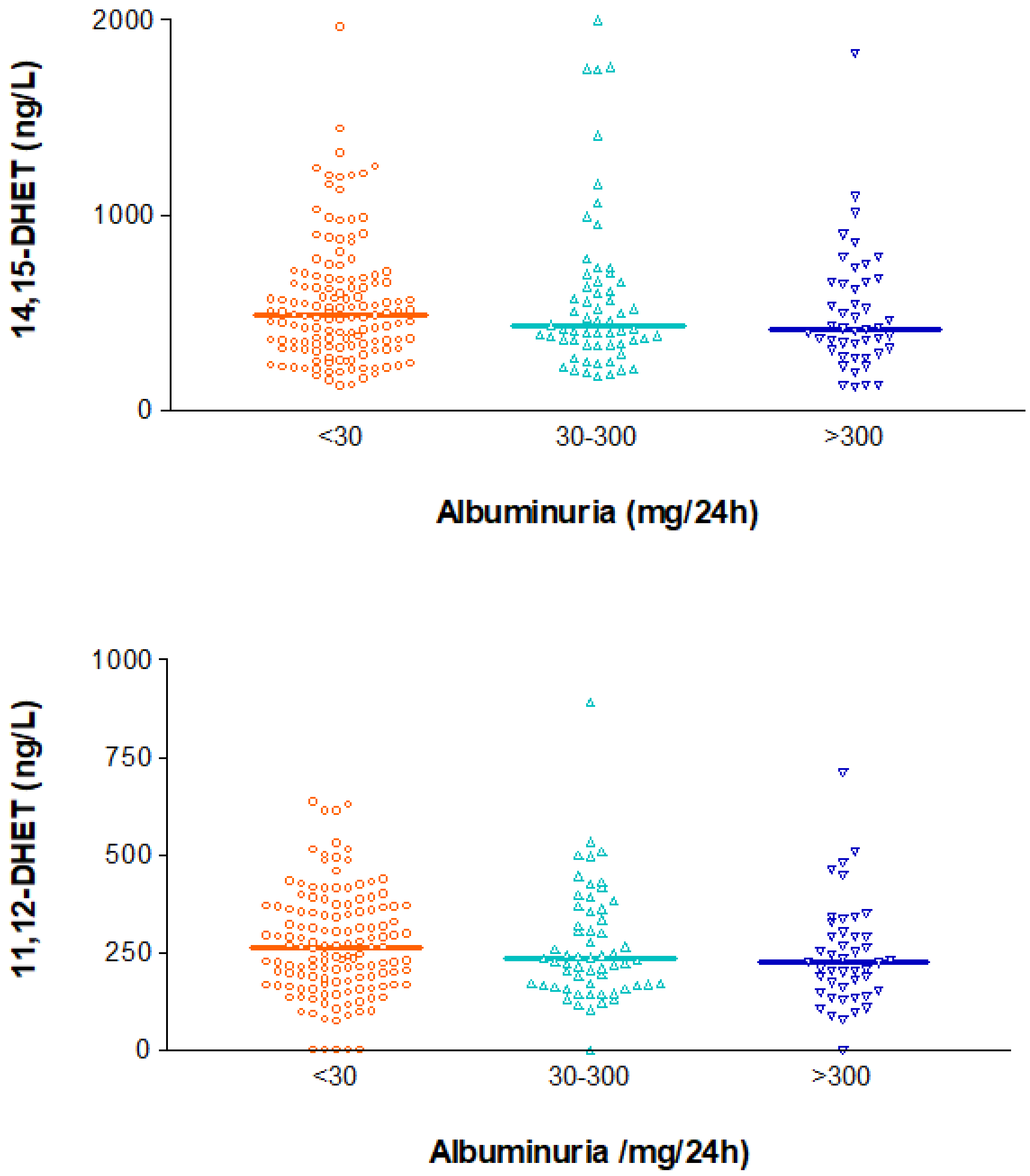

Supplementary Figure 2: Distribution of 14,15- and 11,12-DHET plasma levels according to albuminuria in diabetic and non-diabetic subjecs

EXCLI Journal 2021;20:698-708 – ISSN 1611-2156
Received: January 18, 2021, accepted: March 11, 2021, published: March 18, 2021

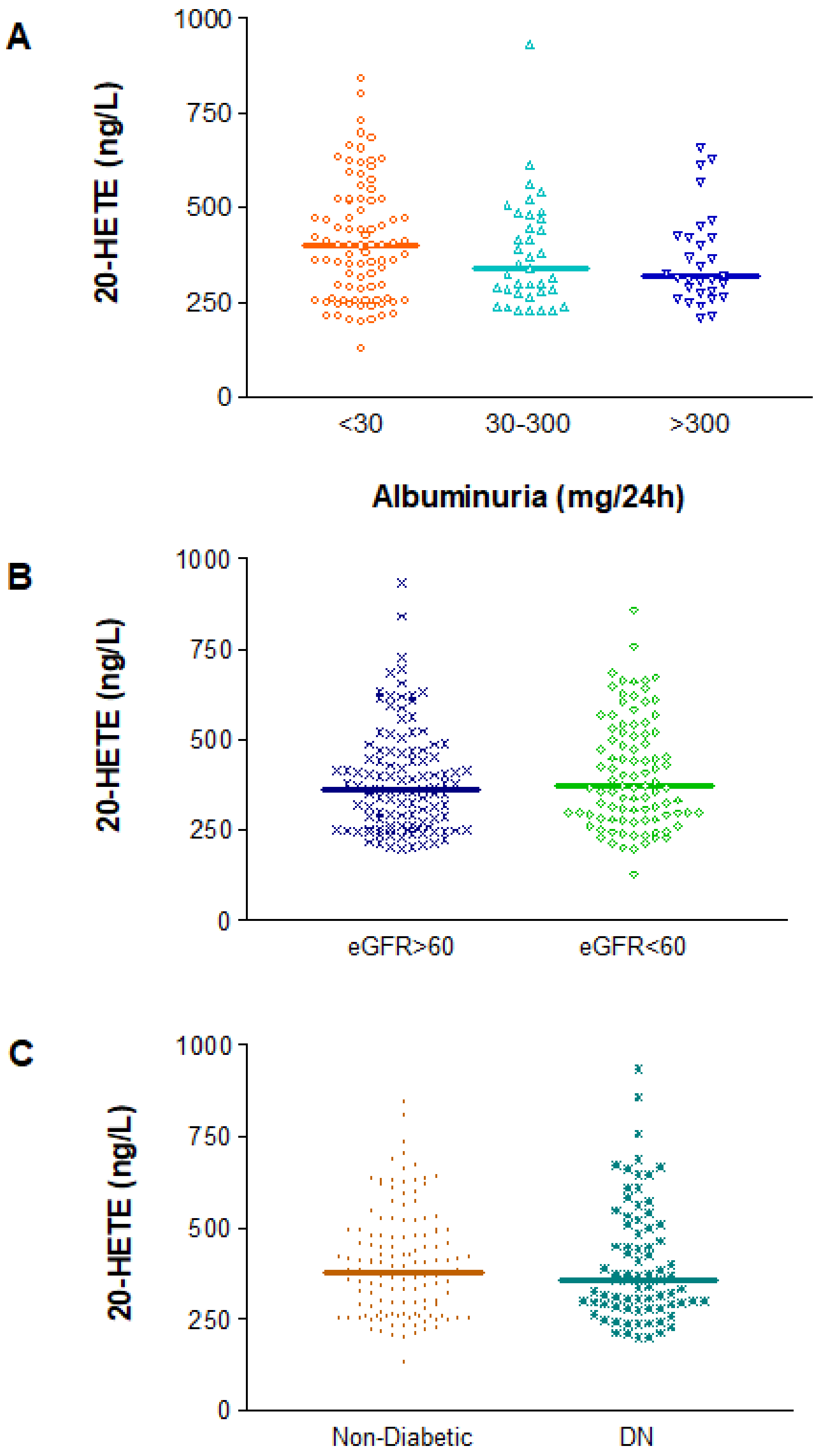

Supplementary Figure 3: Distribution of 20-HETE plasma levels according to abuminuria (**A**), glomerular filtration rate (eGFR) (**B**), and association with diabetic nephropathy (**C**)
